# Meta-analyses are no substitute for registered replications: a skeptical perspective on religious priming

**DOI:** 10.3389/fpsyg.2015.01365

**Published:** 2015-09-15

**Authors:** Michiel van Elk, Dora Matzke, Quentin F. Gronau, Maime Guan, Joachim Vandekerckhove, Eric-Jan Wagenmakers

**Affiliations:** ^1^Department of Psychology, University of AmsterdamAmsterdam, Netherlands; ^2^Cognition and Individual Differences Lab, Department of Cognitive Sciences, University of California at IrvineIrvine, CA, USA

**Keywords:** religious priming, prosocial behavior, meta-analysis, PET-PEESE, Bayesian Bias Correction, preregistration

## Abstract

According to a recent meta-analysis, religious priming has a positive effect on prosocial behavior (Shariff et al., 2015). We first argue that this meta-analysis suffers from a number of methodological shortcomings that limit the conclusions that can be drawn about the potential benefits of religious priming. Next we present a re-analysis of the religious priming data using two different meta-analytic techniques. A Precision-Effect Testing–Precision-Effect-Estimate with Standard Error (PET-PEESE) meta-analysis suggests that the effect of religious priming is driven solely by publication bias. In contrast, an analysis using Bayesian bias correction suggests the presence of a religious priming effect, even after controlling for publication bias. These contradictory statistical results demonstrate that meta-analytic techniques alone may not be sufficiently robust to firmly establish the presence or absence of an effect. We argue that a conclusive resolution of the debate about the effect of religious priming on prosocial behavior – and about theoretically disputed effects more generally – requires a large-scale, preregistered replication project, which we consider to be the sole remedy for the adverse effects of experimenter bias and publication bias.

Does religion make nicer people? The answer to this important question bears on fundamental views about the evolution of religion and the adaptive value of supernatural beliefs (Norenzayan et al., 2014). Evolutionary accounts of religion have proposed that belief in supernatural agents is either a by-product of ordinary cognitive faculties, or that religious beliefs directly confer an adaptive advantage (Pyysiäinen and Hauser, 2010). According to the latter view, beliefs in an invisible supernatural agent who rewards good behaviors and who punishes moral transgressions provided a solution to the “freerider problem”: individuals may try to obtain the benefits from being considered a group member without contributing to the group. When societies increased in size, the freerider problem became more apparent because it was impossible to track the behavior of all individuals in the group. Accordingly, it is pointed out that the belief in “moral gods” first appeared when people started living in city-states. The continuous feeling of being watched by a supernatural agent enforced moral and prosocial behavior.

The adaptationist view of religious beliefs is gaining more and more empirical support as a result of studies showing a relation between religion and prosociality. When using self-report measures, religious participants typically report stronger prosocial attitudes and more prosocial behavior than non-religious participants. However, when it comes to actual behavioral measures (e.g., performance in economic decision games) the answer is less straightforward: religious participants appear equally altruistic (or egoistic) as their non-religious counterparts, and if they donate money at all, they do so preferably to ingroup members (Galen, 2012).

Conceptual priming has been introduced as an important methodological innovation to overcome problems inherent to self-report measures (Bargh, 2006). In an influential paper, Shariff and Norenzayan (2007) found that when participants were implicitly primed with religious concepts, they donated more money in an economic decision game. This effect has been interpreted as supporting the “supernatural monitoring hypothesis”: the activation of religious concepts induces the feeling of a supernatural agent that monitors our behavior (Norenzayan et al., 2014). In turn, the feeling of “being watched” fosters moral and prosocial behavior.

Following discussions on the reliability of conceptual priming techniques and the potential file-drawer problem in the field (e.g., Francis, 2012), Shariff et al. (2015) have recently conducted a meta-analysis on the effects of religious priming. The authors conclude that (1) when taking into account both published and unpublished studies, religious priming exerts a small though reliable effect on behavioral measures; (2) religious priming is mainly effective for religious participants (i.e., religiosity is an important moderator of the priming effect); and (3) explicit priming techniques (e.g., using a contextual manipulation, such as conducting the experiment in a church or presenting participants with the ‘call for prayer’) result in stronger effects than implicit priming techniques (e.g., using subliminal primes or a scrambled sentence task).

These findings may seem to support the notion of religious priming in particular and the use of conceptual priming techniques in general, and Shariff et al. (2015) are to be commended for their effort in taking this important initiative. However, despite the authors’ best efforts, several methodological and statistical concerns remain. In our opinion, these concerns strongly limit the inferences that may be drawn from this meta-analysis. Moreover, we re-analyzed the Shariff et al. (2015) data using two alternative meta-analytic techniques that seek to correct for publication bias [i.e., the Precision-Effect Testing–Precision-Effect-Estimate with Standard Error (PET-PEESE) method and a Bayesian bias correction (BBC) method]. These two alternative meta-analyses yield opposing conclusions, a result that reinforces our opinion that meta-analyses alone cannot conclusively establish the reliability and replicability of theoretically disputed effects (see also Wagenmakers et al., 2015). The main reason for our skepticism, however, is that overall effect size estimates from a meta-analysis result from the combination of a possible true effect and a spurious effect that reflects the influence of both publication bias and experimenter bias (e.g., Barber, 1976; John et al., 2012). To establish the robustness of psychological effects without the confounding influence of such biases, we argue for the use of large-scale and preregistered replication attempts, as facilitated for instance by the Open Science Framework (OSF).

## Methodological and Statistical Concerns

Here we list five concerns with the meta-analysis conducted by Shariff et al. (2015), all of which we believe reduce the evidential impact of the results. First, the authors report a negative correlation between sample size and effect size, with high-powered studies showing an effect size that hovers around zero. The authors note that almost all meta-analyses show a similar negative relation between sample size and effect size; to us, this suggests that the results are contaminated by publication bias (e.g., the tendency to report low-N studies only when these yield a significant result) and/or questionable research practices (QRPs; e.g., optional stopping when the *p*-value approaches 0.05). Note that the observed association between sample size and effect size alone does not allow one to determine the relative contribution of publication bias and/or QRPs. Thus, our main concern is that studies with larger sample sizes tend to provide a more accurate estimate of the true effect size, and that these estimates are close to zero.

It should be acknowledged that the authors used the ‘trim-and-fill’ method to correct for publication bias. However, as argued in detail elsewhere (Simonsohn et al., 2014), this method rests on the unlikely assumption that selective reporting is related to researchers being hesitant to report small effect sizes, whereas in practice results are left unreported because the *p*-value does not reach the critical threshold of 0.05. Below we report the outcomes of two alternative meta-analytic procedures, namely PET-PEESE (e.g., Stanley and Doucouliagos, 2014) and the BBC method (Guan and Vandekerckhove, 2015) to correct for publication bias.

A second concern is that Shariff et al. (2015) may have relied on overly selective inclusion criteria and have failed to specify the reasons why specific (unpublished) studies were excluded from the final analysis. By only including a select subset of studies, the observed effect size may be severely overestimated. For instance, the Appendix of the Shariff et al. (2015) meta-analysis lists studies that were excluded because no ‘neutral’ prime condition was included, but it is unclear how this criterion was defined (e.g., why should a ‘sports prime’ be more neutral than a ‘student prime’ or an ‘animal prime’ condition?). Moreover, a quick online search reveals that at least 14 unpublished manuscripts reporting studies on religious priming could have been included in the meta-analysis (see the Appendix to the present paper). All these manuscripts were available before 2014. Of these 14 studies, four report positive evidence for religious priming, eight report mixed evidence (i.e., the effect is only observed for a specific sub-group such as males or the effect is in an unexpected direction), and two report a null-result.

Our third concern relates to the analysis of the possible moderator of religiosity. Shariff et al. (2015) note that the small effect sizes for religious priming effects on prosocial behavior may be related to the (lack of) religiosity of the participants involved in the studies. In support of this hypothesis, the authors report a stronger effect size for religious compared to non-religious participants. However, the concerns mentioned above apply even more to the small subset of studies that were included in this “religiosity-as-moderator” analysis: a strong negative relation between effect size and sample size and an incomplete selection of studies. For instance, we recently reported six experiments showing a null-effect of religious priming for both religious and non-religious participants (Van Elk et al., 2014; a paper excluded from the meta-analysis).

A fourth concern is that Shariff et al. (2015) did not control for important and well-established determinants of prosocial behavior such as gender, education, and socio-economic status (cf. Eagly, 2009; Piff et al., 2010). It should be noted that the meta-analysis was based on studies in which participants were randomly assigned to the experimental or the control condition. We also note that controlling for demographic variables in a meta-analysis may be difficult. Nevertheless, as we argue below, pre-registered replication studies should attempt to control for important determinants of prosocial behavior and investigate the extent to which religious priming exerts an additional effect beyond individual differences in prosocial behavior.

**FIGURE 1:**
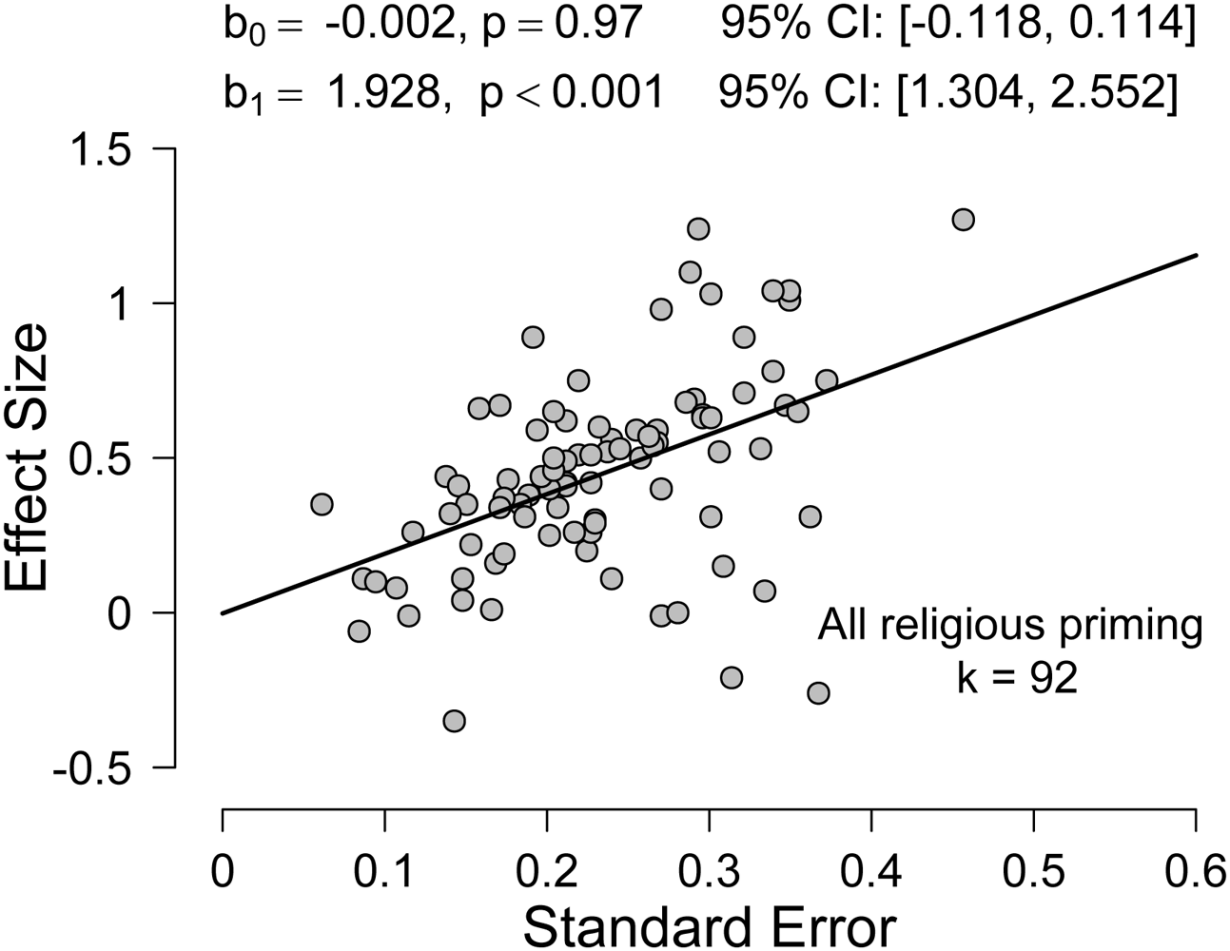
**Fit of the Precision-Effect Testing (PET) model to all religious priming studies (*k* = 92) in the meta-analysis by Shariff et al. (2015)**.

A fifth concern is evident from a consideration of the forest plot depicted in Shariff et al. (2015; **Figure 1**). The forest plot shows that the confidence intervals (CIs) for the effect sizes of the individual studies are quite large (mean width of CI = 0.92), whereas, at the same time, for the majority of studies the lower bound of the CI lies just above zero (mean range of CI = [0.0–0.91]); for instance, the CI of 35% of the studies is in the interval [0.0–0.1]. Given the uncertainty in the estimation of the CI, such a pattern is statistically unlikely and – similar to the association between sample size and effect size discussed above – it suggests biased reporting of the observed effects (Schimmack, 2012; Francis, 2013; but see: Morey, 2013; Simonsohn, 2013). In order to obtain an alternative to the ‘trim-and-fill’ estimate of effect size in the presence of publication bias and selective reporting, we applied the PET-PEESE (e.g., Stanley and Doucouliagos, 2014) and the BBC method (Guan and Vandekerckhove, 2015) to the effect sizes reported by Shariff et al. (2015). The outcomes of these analyses are described below.

## Re-Analysis I: The PET-PEESE Method

The PET-PEESE procedure involves two regression models, PET (Stanley, 2005) and PEESE (Stanley and Doucouliagos, 2007) and a rule to decide which of the two models should be used^1^.

The PET model (Stanley, 2005) is based on Egger’s regression test (Egger et al., 1997) and regresses the observed effect sizes on their corresponding SE. The intuition is as follows: in the absence of publication bias, there is no relation between the SE of a study and the reported effect size; consequently, a plot with SE on the x-axis and reported effect size on the y-axis will show a random scattering of points around a horizontal line that corresponds to the true underlying effect size. The variance of this scattering depends on the SE: effect sizes from studies with small SE will cluster closely around the true value, but, as the SE increases, so does the variance of the observed effect sizes. With regard to the regression model, this inherent heterogeneity of error variance necessitates a weighted least squares regression model where the weights correspond to the inverse of the squared SE.

In the presence of publication bias, however, the pattern of results will be different. Publication bias means that non-significant results will tend not to be published, and this results in a positive correlation between SE and effect sizes: low-N studies have a relatively large SE, and they require larger effect sizes to obtain significant results. In the PET model, the slope coefficient quantifies publication bias (i.e., a positive slope indicates that publication bias is present) and the intercept coefficient estimates the true effect corrected for publication bias (i.e., the intercept corresponds to an idealized study with “zero” SE).

The PEESE model (Stanley and Doucouliagos, 2007) differs from the PET model in that it regresses the effect sizes on the squared SE. The PEESE model performs better in the presence of a true effect, whereas the PET model performs better in the absence of a true effect (e.g., Stanley and Doucouliagos, 2014). Consequently, Stanley and Doucouliagos (2014) proposed to use the intercept of the PET model as the estimate of the true effect whenever this intercept is non-significant and to use the intercept of the PEESE model otherwise.

**Figure 1** shows the results of applying the PET-PEESE procedure to the 92 religious priming studies analyzed by Shariff et al. (2015). The coefficients for PET were *b_0_* = –0.002, *p* = 0.97, 95% *CI* [–0.118, 0.114] and *b_1_* = 1.982, *p* < 0.001, 95% *CI* [1.304, 2.552]. Since the intercept *b_0_* was not significantly different from zero, the PET-PEESE estimate of the true underlying effect is –0.002. Furthermore, the significant positive slope *b_1_* suggests the presence of publication bias.

**FIGURE 2:**
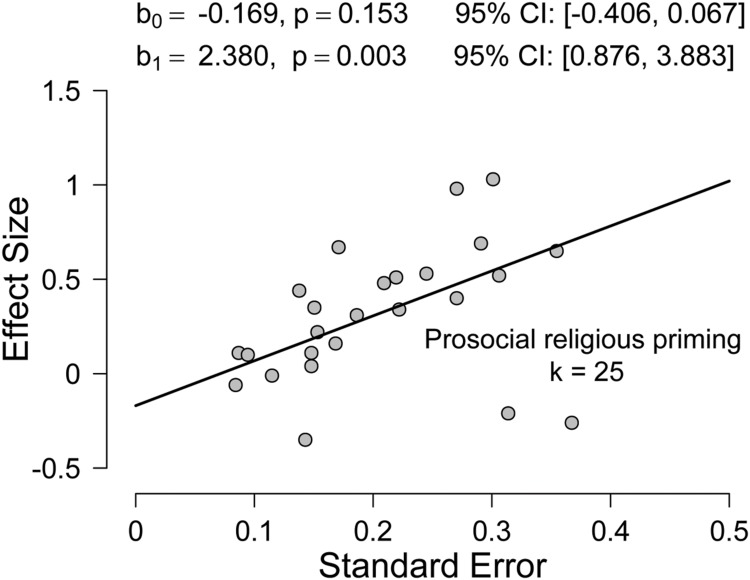
**Fit of the PET model to the subset of the religious priming studies explicitly focusing on the priming of prosocial behavior (*k* = 25) in the meta-analysis by Shariff et al. (2015)**.

**Figure 2** shows the result of applying the PET-PEESE procedure to the subset of 25 religious priming studies that investigated effects on prosocial behavior. The coefficients for PET were *b_0_* = –0.169, *p* = 0.153, 95% *CI* [–0.406, 0.067] and *b_1_* = 2.380, *p* = 0.003, 95% *CI* [0.876, 3.883]. Since the intercept *b_0_* was not significantly different from zero, the PET-PEESE estimate of the true underlying effect is –0.169. Again, the significant positive slope *b_1_* suggests the presence of publication bias.

To summarize, when we corrected for publication bias by applying the PET-PEESE method, we were unable to confirm the results reported by Shariff et al. (2015). The PET-PEESE estimates provided no evidence for a positive effect of religious priming on prosocial behavior; if anything, the bias-corrected PET-PEESE effect size is slightly negative.

## Re-Analysis II: The Bayesian Bias Correction Method

An alternative approach that can account for the effects of publication bias was recently developed by Guan and Vandekerckhove (2015). This novel approach is based on a Bayesian procedure that assumes one or several biasing processes may have been at play while the papers were being published. These behavioral processes are then quantified, and Bayesian inference is applied to estimate the probability that each of the processes was active. Conditional on each process, an updated effect size is computed, and these effect sizes are finally averaged with the model probabilities as weights (a procedure known as Bayesian model averaging, e.g., Hoeting et al., 1999).

We applied the BBC method to the effect size estimates reported by Shariff et al. (2015). In order to conduct a Bayesian analysis, we are required to make explicit our prior assumptions regarding the relative likelihood of effect sizes. For the current analysis, we used as prior a normal distribution centered on 0, with SD equal to 2/3 (which implies that effect sizes are equally likely to be less or greater than 0.45 in absolute value). We note that although the Bayes factor is somewhat sensitive to the exact definition of the prior, the results in this case were qualitatively the same for all prior SD within a reasonable range (we tested SDs as small as 1/10). Furthermore, it should be noted that the BBC method is currently limited to homogeneous underlying effect sizes, a limitation that may be particularly pressing in the current context.

**FIGURE 3:**
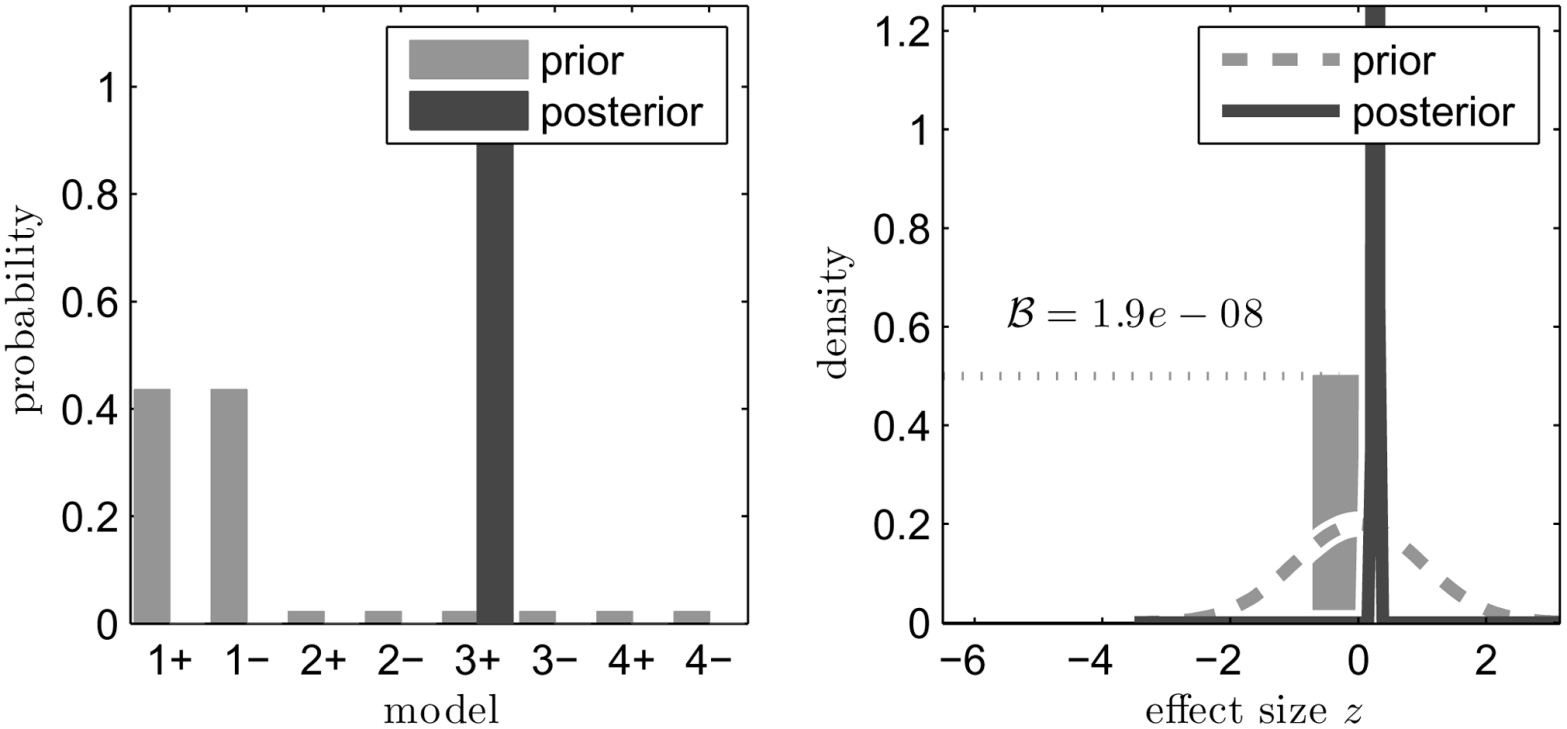
**Results of the Bayesian bias correction (BBC) reanalysis of the religious priming studies.** In the **(left)**, we display the prior (gray) and posterior (black) probabilities of eight possible scenarios that result from the combination of four possible bias processes and two states of nature (to wit, that there is a real effect [+] vs. that there is no effect [–]). The greatest positive change, and the largest resulting posterior probability, is seen in model 3+. This model includes a biasing process whereby non-significant results are published at a smaller, but nonzero rate. Additionally, it includes a true effect. The high posterior likelihood on this model implies that a true effect exists. In the **(right)**, we display the prior and posterior distributions of this effect size. The gray dashed line and gray bar indicate the prior distribution of the effect size, with a 50% probability of it being exactly 0 and a 50% probability of it being somewhere in the domain of the normal distribution. In the (black) posterior distribution, the point mass at 0 has vanished as the mass of the posterior has accumulated around 0.3. As a result, the Bayes factor favoring the null hypothesis is practically 0, indicating strong evidence for the alternative hypothesis that there is an effect of religious priming.

The outcome of the BBC diverged from that of PET-PEESE. In the full set of religious priming studies, BBC indicated the presence a real effect in addition to the presence of publication bias (see **Figure 3**). In the prosocial subset, BBC indicated the presence of a real effect and very little evidence for publication bias. In both cases, the evidence for the existence of a real effect was judged to be very strong.

## Limitations of a Meta-Analysis

As the outcomes from our re-analyses demonstrate, different meta-analytic techniques can lead to opposite conclusions. According to the BBC and the trim-and-fill method, religious priming indeed makes people more prosocial. In contrast, according to the PET-PEESE method, there is no effect of religious priming on prosocial behavior. So what can we conclude from all this? We would like to highlight two points before ending with a general recommendation for future studies on religious priming.

First, as discussed above, the Shariff et al. (2015) meta-analysis suffers from selection bias with respect to the studies that were included. In addition, all meta-analytic methods converged on the conclusion that publication bias indeed plagues the field of religious priming. The different meta-analytic techniques rely on different assumptions and by using alternative ways of correcting for publication bias, they may lead to different bias-corrected effect size estimates. The choice for one method over another may be guided by theoretical considerations (most of the authors of the current paper have a preference for the Bayesian method), but because the true extent of publication bias is unknown, the question as to which method provides the most accurate estimate of the true effect size remains open.

Second, in the absence of preregistration, the outcome of each individual study that was included in the meta-analysis is prone to QRPs (e.g., John et al., 2012), experimenter expectancy effects, biases due to motivated reasoning, and biases due to optional stopping during data collection (i.e., continuing the analysis until the desired result is found). Such biases can express themselves through selection procedures such as excluding participants that do not show the effect, reporting only those measures that show an effect, promoting pilot experiments to main studies whenever the initial results are promising, etc. (e.g., De Groot, 1956/2014). Thus, when a meta-analysis is conducted on studies that have not been preregistered, the resulting effects are a mix of possible true effects and effects that are due to experimenter bias. Scientists are prone to many implicit and explicit cognitive biases that skew their methods and results (Podsakoff et al., 2003). A meta-analysis relies on the observed effect sizes, and cannot disentangle effects that are real from those that are the result of human bias in reporting and analysis. Hence, a meta-analysis cannot provide compelling evidence for the presence of a disputed effect if the individual studies are prone to experimenter bias effects. In other words, if there is a true effect, then a meta-analysis will pick it up. But a meta-analysis will also indicate the existence of an effect if there is no true effect but only experimenter bias and QRPs, which result in Type I error inflation and the overestimation of effect sizes.

With respect to religious priming, we argue that the Shariff et al. (2015) meta-analysis cannot provide the evidence that is required to convince the skeptic that religious priming exists. The significant result that was obtained by combining the data from many studies may merely reflect the fact that some proportion of these experiments suffer from publication and experimenter bias to an unknown degree.

## Recommendation

In our opinion, the only compelling remedy against the pervasive problem of experimenter bias and publication bias is the use of large-scale preregistered replication attempts such as those supported by the OSF and the Registered Replication Reports initiative (Simons et al., 2014; see also Chambers, 2013; Nosek and Lakens, 2014). We do not, however, wish to convey the impression that we believe meta-analyses are useless. For scientific purposes, meta-analyses can be highly informative, even when the underlying studies are prone to experimenter bias. Again, we would like to compliment Shariff et al. (2015) for their tremendous effort in taking the issue of religious priming seriously. Although we have highlighted several concerns, the Shariff et al. (2015) meta-analysis provides a valuable starting point and recommendations for future studies with respect to sample size and potential moderators.

First, Shariff et al. (2015) provide an effect size estimate for religious priming, which can be used to guide future research. Starting from the most optimistic effect size estimate reported in the meta-analysis (i.e., *g* = 0.40; reflecting the overall effect of religious priming uncorrected for publication bias across all studies irrespective of the dependent measure), a simple calculation indicates that in order to achieve 80% power in a between-subject design (experimental vs. control condition), one needs to test a total of 156 participants. When prosocial behavior is used as dependent variable, the effect size estimate (corrected for publication bias) is *g* = 0.18, which, in order to obtain 80% power, requires a total of 766 participants to detect an effect^2^. This demonstrates that many studies in the field of ‘religious priming’ research are severely underpowered. Future studies would do well to use sufficiently large samples in order to demonstrate an effect of religious priming, preferably by using preregistration and collaboration between different labs.

Second, the meta-analysis by Shariff et al. (2015) suggests that religious priming works selectively for religious participants and when explicit rather than implicit priming methods are used. These are interesting and concrete suggestions that call for a direct, confirmatory test in a many-labs replication study; ideally, such a study also takes into account the potential moderating role of cultural context (e.g., secular vs. religious countries) and controls for other moderators, such as gender, socio-economic status, and income.

Interestingly, a recent high-powered and pre-registered replication attempt of study 2 of Shariff and Norenzayan (2007) showed a null-result: religious priming did not result in more prosocial behavior (Gomes and McCullough, 2015). In addition, a pre-registered study on religious priming on prosocial behavior from our own lab, using two large samples of highly religious Dutch and US participants also failed to show an effect (Maij and van Elk, in preparation). Note, however, that these studies differed from the original study in terms of experimental design and procedure. Future studies should aim to systematically determine the potential boundary conditions of religious priming effects on prosocial behavior. We hope that the original authors and other researchers will participate in such an endeavor, possibly in the form of a many-labs adversarial collaboration (e.g., Matzke et al., 2015). Only after eliminating experimenter bias and researcher’s degrees of freedom, can we establish whether religion indeed makes nicer people.

## Conflict of Interest Statement

The authors declare that the research was conducted in the absence of any commercial or financial relationships that could be construed as a potential conflict of interest.

## Footnotes

1 We thank Daniel Lakens for calling our attention to the advantages of the PET-PEESE analysis. For a recent critical discussion on the limitations of the PET-PEESE method, see http://willgervais.com/blog/2015/6/25/putting-pet-peese-to-the-test-1.

2 Only one study in the meta-analysis actually meets this criterion, but in that study no overall religious priming-effect was observed (i.e., religious priming increased prosocial behavior for Protestant participants, decreased prosocial behavior for Catholic participants, and did not affect prosocial behavior for participants coming from a different religious denomination; Benjamin et al., 2010).

## APPENDIX

Studies on religious priming retrieved from the internet that were not included in the Shariff et al. (2015) meta-analysis.

Badi, J. A., and Ayinde, O. L. (2013). Effects of Priming on Muslims’ Behaviors: an empirical study. *Int. J. Appl. Sci. Technol.* 3, 21–27.

Bloom, P. B., and Arikan, G. (2013). Priming religious belief and religious social behavior affects support for democracy. *Int. J. Public Opin. Res.* 25, 368–382.

Bowen, D., and Cheng, A. (2014). Peering into the black box of faith-based education: do religious cues affect self-regulation and political tolerance? *EDRE Working Paper NO 2014-01*.

Burson, A. (2007). *Self-Regulation and Religiosity*. Ph.D. thesis, Ohio State University, Columbus, OH.

Card, S. (2013). *The Effects of the Activation of Differentiated Concepts of God on Prosocial Behavior*. Master thesis, Utrecht University, Utrecht.

Carter, E. C. (2010). *Religious Cognition and Duration of Maintained Grip*. Open Access thesis, Paper 23. University of Miami, Coral Gables, FL.

Hone, L. S. E., and McCullough, M. E. (2014). Does religious cognition really down-regulate hand grip endurance in men? A failure to replicate. *Evol. Hum. Behav.* 36, 81–85.

Norry, C. (1987). *The Effect of Priming, Christian Orthodox Beliefs, and Training on Critical Thinking*. Thesis and dissertations, Paper 524. Wilfrid Laurier University, Waterloo, ON.

Parra, J. C. (2011). *The Effects of Religion on Social Cooperation: Results from a Field Experiment in Ghana*. Working Paper. Georgetown University, Washington, DC.

Qin, C. K. (2011). *Taking a Leap of Faith: Reminders of God Lead to Greater Risk Taking*. Master thesis, National University of Singapore, Singapore.

Salomon, E. C. (2013). *The Effect of Religious Priming on Recalled Regret*. Master thesis, University of Illinois, Champaign, IL.

Stachtiaris, S., Drichoutis, A., Nayga, R., and Klonaris, S. (2011). Can religious priming induce truthful preference revelation? *MPRA Paper No. 34433.*

Tidwell, J. (2011). *The Effect of Persuasive Appraisals after Priming Faith and Trust in God*. Master thesis, University of Houston, Houston, TX.

Vilaythong, T., Lindner, N. M., and Nosek, B. A. (2010). “Do Unto Others”: effects of priming the Golden Rule on Buddhists’ and Christians’ attitudes toward gay people. *J. Sci. Study Relig.* 49, 494–506.
